# The Involvement of Glutamate-mGluR5 Signaling in the Development of Vulvar Hypersensitivity

**DOI:** 10.3390/ijms26020523

**Published:** 2025-01-09

**Authors:** Yaseen Awad-Igbaria, Saher Abu-Ata, Reem Sakas, Sarina Bang, Tom Fishboom, Alon Shamir, Jacob Bornstein, Lior Lowenstein, Eilam Palzur

**Affiliations:** 1 Azriele Faculty of Medicine in the Galilee, Bar-Ilan University, Safed 1311502, Israel; saher9644@gmail.com (S.A.-A.); reem_sakas@hotmail.com (R.S.); tomf.tbi.lab009@gmail.com (T.F.); liorl@gmc.gov.il (L.L.); mdjacob@gmail.com (J.B.); 2 Research Institute of Galilee Medical Center, Nahariya 2210001, Israel; 3 Department of Obstetrics and Gynecology, Galilee Medical Center, Nahariya 2210001, Israel; 4 Psychobiology Research Laboratory, Mazor Mental Health Center, Akko 2412001, Israel; alon.shamir9@gmail.com; 5 Ruth and Bruce Rappaport Faculty of Medicine, Technion—Israel Institute of Technology, Haifa 3478403, Israel

**Keywords:** provoked vulvodynia, inflammation, neuromodulation, central sensitization, mGluR5

## Abstract

Provoked vulvodynia (PV) is the leading cause of vulvar pain and dyspareunia. The etiology of PV is multifactorial and remains poorly understood. PV is associated with a history of repeated vulvar inflammation and is often accompanied by sensory neuromodulation as a result of activation of the metabotropic glutamate receptor 5 (mGluR5) in the sensory nerve terminals. Therefore, this study aims to examine the role of glutamate-mGluR5 signaling during the initial inflammatory phase in chronic vulvar pain development in an animal model of PV.Thermal and mechanical vulvar sensitivity was assessed for three weeks following zymosan vulvar challenges. Anxiety-like behavior and locomotor activity were assessed at the end of the experiment. To investigate the role of glutamate mGluR5, the MTEP (mGluR5 antagonist) was injected into the vulva during vulvar inflammation. On the other hand, glutamate or CHPG (mGluR5 agonist) were injected in order to examine the effects of mGluR5 activation. RT-PCR was performed to assess changes in the transcription of genes related to neuroinflammation, neuromodulation, and neuroplasticity in the spinal cord (L6-S3). Zymosan-induced inflammation resulted in a significant thermal and mechanical vulvar hypersensitivity that persisted for over a month after the zymosan injection. However, local treatment with MTEP enhanced the vulvar mechanical and thermal hypersensitivity. On the other hand, activation of the mGluR5 via injection of glutamate or CHPG into the vulva leads to long-lasting vulvar mechanical and thermal hypersensitivity. The activation of the glutamate pathway was found to be accompanied by an increase in the transcription level of genes related to neuroinflammation and neuroplasticity in the sacral spine region. The present findings indicate that vulvar hypersensitivity is mediated by mGluR5 activation during inflammation. Hence, modulation of the mGluR5 pathway during the critical period of inflammation contributes to preventing chronic vulvar pain development. Conversely, activation of the mGluR5 pathway leads to long-lasting mechanical and thermal hypersensitivity.

## 1. Introduction

Vulvodynia is chronic vulvar pain lasting at least 3 months [1,2]. The most common type of vulvodynia is “provoked vulvodynia” (PV), which is characterized by localized hypersensitivity and severe pain of the vulvar vestibule following stimulation [1,2,3]. PV affects 7–15% of women of all ages [1], and it is linked with psychological and mental health disorders including anxiety, depression, reduced sexual desire, and sexual satisfaction [4,5,6,7,8].

The pathogenesis and etiology of PV remain poorly understood, complex, and uncertain [9,10]. Previous findings indicate that PV might be associated with vulvar microbial changes [11,12,13], genetic predisposition [14,15,16,17,18], and hormonal and immunological factors [2,19,20,21,22,23,24]. Yet a substantial body of evidence indicates that PV is associated with repeated vulvar inflammation. The main hypothesis is that repeated vestibular inflammation may lead to long-lasting neuronal adaptations and remodeling, causing vulvar hypersensitivity [25,26,27,28,29,30,31].

According to the inflammatory theory, in response to inflammatory stimuli, immune and damaged cells at the inflammation site discharge various mediators including nerve growth factor (NGF), pro-inflammatory cytokines, and neurotransmitters such serotonin and glutamate that are probably involved in nerve sensitization [26,29,30,32]. Interestingly, previous evidence has shown that, during peripheral inflammation, the activation of the glutamate signaling pathway plays a central role in pain signaling and hypersensitivity [33]. Thus, the released glutamate from the inflamed tissue site can activate the metabotropic glutamate receptor 5 (mGluR5) in the sensory nerve terminals, causing the sensitization of nociceptors including the transient receptor potential cation channel vanilloid and ankyrin 1 (TRPV1 and TRPA1), which are responsible for thermal and mechanical sensation [33]. Conceivably, the stimulation of the glutamate pathway in the vulva can activate the secondary signaling in neurons, leading to short-term modifications such as phosphorylation of the ion pain channels, resulting in a lower pain threshold [26]. Previous findings support this assumption. Thus, activation of the mGluR5 in sensory neurons elevates intracellular signaling pathways, such as protein kinase signaling, which contribute to the upregulation of TRP channel expression and increased neuronal excitability [33].

More critically, repeated activation of the glutamate signaling pathway in the neurons might lead to long-lasting changes in the sensory pathway, such as overexpression of the pain channels, neuroinflammation, neuromodulation, and neuroplasticity in the vulvar nerves and spinal cord [33,34,35,36]. Thus, modulating the glutamate signaling pathway in the vulva during the inflammation might be an effective strategy to prevent the development of chronic vulvar pain. Additionally, the heightened understanding of the glutamate signaling in pain development will give rise to new hypotheses, opening opportunities to develop pioneering proof-of-concept strategies aimed at addressing vulvar pain development and maintenance. Accordingly, the aim of the present study is to examine the role of the glutamate-mGluR5 signaling pathway during repeated vulvar inflammation in vulvar hypersensitivity development.

## 2. Result

### 2.1. Modulating Vulvar Hypersensitivity Development by Interrupting the Glutamate-mGluR5 Pathway During Inflammation

**Figure 1:**
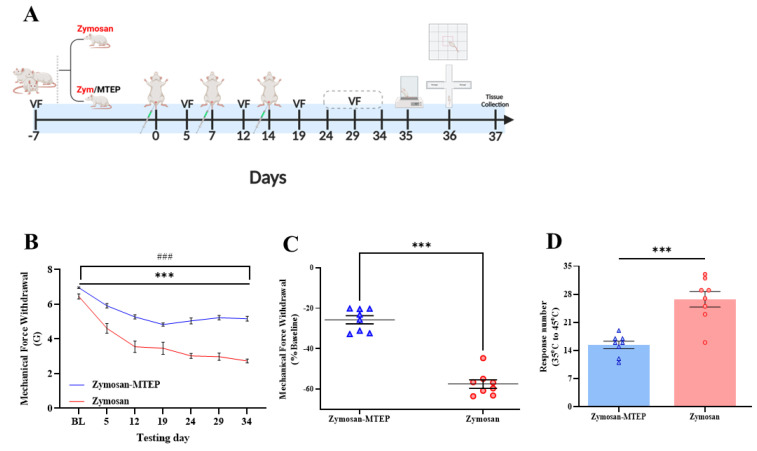
Blocking glutamate-mGluR5 signaling during the vulvar inflammation modulates the development of vulvar hypersensitivity. (**A**) The experimental timeline and the experimental procedures across three challenges of vulvar inflammation. MTEP was injected into the vulva 4, 24, and 48 h after each zymosan injection. Vulvar MST was measured by Von Frey (VF). Hot plate tests, EPM tests, and open field tests were performed on day 35 and 36. (**B**) Vulvar mechanical force withdrawal (G) of the zymosan and zymosan/MTEP groups. (**C**) Vulvar mechanical force withdrawal test on day 34, normalized to the baseline. (**D**) Sum of nociceptive responses in the hot plate test that was performed on day 35. Behavioral assessment (*n* = 8 per group). Mixed model ANOVA and student’s *t*-tests were performed. Mean ± SEM. ### *p <* 0.001 compared to baseline; *p <* 0.005, *** *p <* 0.001. Difference between groups.

Inflammation promotes the upregulation of glutamate-mGluR5 pathways, contributing to neuromodulation and neuroplasticity [26,33]. Consequently, we speculate that blocking glutamate-mGluR5 signaling pathways will prevent chronic vulvar pain development. To examine this hypothesis, MTEP-mGluR5 antagonist (100 µL of 10 mM SC in vulvar) was injected during the early inflammation period (4, 24, and 48 h after zymosan injection) (Figure 1A).

In both groups, i.e., zymosan and zymosan-MTEP groups, there was a significant reduction in the vulvar mechanical threshold (Figure 1B). However, the local treatment by MTEP (100 µL of 10 mM) proved to be beneficial in modulating the development of chronic vulvar pain. Thus, five days after each challenge, a significant difference in MST was observed between the zymosan group and the zymosan/MTEP group. In the 1st inflammation challenge, both groups recovered to the non-allodynic levels (Figure 1B). In the 2nd vulvar challenge, 85% of the rats in the zymosan/MTEP group recovered to the non-allodynic levels, compared to 12.5% in the zymosan group. In the 3rd challenge, 12% of the rats in the zymosan/MTEP group recovered to the non-allodynic levels. Furthermore, there was a recovery trend to the non-allodynic levels; thus, at the end of the experiment (20 days after the 3rd inflammation challenge), 62% of the rats in the zymosan/MTEP group showed a recovery to the non-allodynic levels, while no evidence of healing or recovery to the non-allodynic levels was observed in the zymosan group (Figure 1C).

Additionally, a significant difference was observed with respect to the thermal sensitivity test between the zymosan and zymosan/MTEP groups (t_(14)_ = 5.28, *p* < 0.001, Figure 1D). Thus, the zymosan/MTEP group showed a reduction in the thermal sensitivity response, including vulvar licking and rearing, compared to the zymosan group (Figure 1D), suggesting that the modulation of mGluR5 signaling during the vulvar inflammation can modulate the vulvar pain development.

### 2.2. Modulating Glutamate-mGluR5 Pathway During Inflammation Regulates Anxiety-Like Behavior

**Figure 2:**
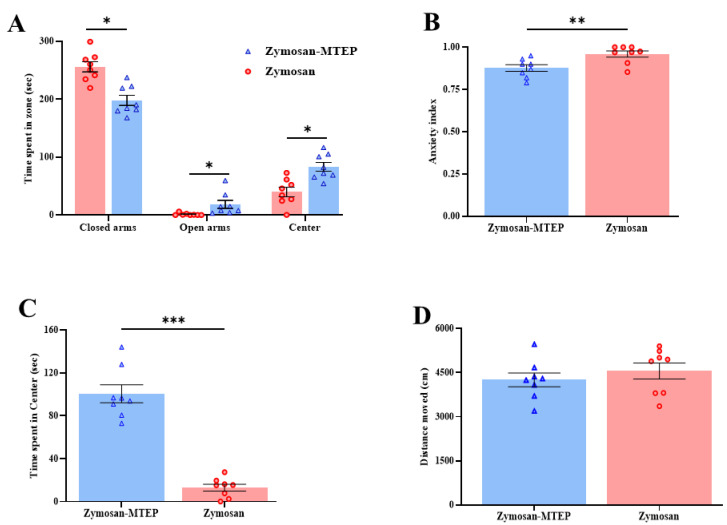
Modulating glutamate-mGluR5 pathway during vulvar inflammation regulates anxiety-like behavior. (**A**) Time spent in the open arms, closed arms, and center of the EPM. (**B**) Anxiety index score of the zymosan and zymosan/MTEP groups (0 = low anxiety score; 1 = high anxiety score). (**C**) Time spent in the center of the open field (second). (**D**) Total distance moved in the open field (cm). Behavioral assessment (*n* = 8 per group). Student’s *t*-tests were performed. Mean ± SEM. * *p <* 0.05, ** *p <* 0.005, *** *p <* 0.001. Difference between groups.

Regarding the anxiety level and locomotor activity, there was a significant difference between the zymosan and the zymosan/MTEP groups in the time spent in the EPM zones (t_(14)_ = 4.63, *p* < 0.001; t_(14)_ = −2.48, *p* < 0.001; t_(14)_ = −3.86, *p* < 0.001, closed arms, open arms, center zone, Figure 2A). Thus, the zymosan/MTEP group spent less time in the closed and more time in the open arms compared to the zymosan group (Figure 2A). In addition, the analysis of the anxiety index score revealed a significant difference between the groups (t_(14)_ = 3.10, *p* = 0.008, Figure 2B). Thus, the zymosan/MTEP group showed a lower anxiety index score compared to the zymosan group (Figure 2B).

In the open field test, there was a significant difference in the time spent in the center zone of the arena between groups (t_(14)_ = −9.68, *p* < 0.001, Figure 2C). Thus, the zymosan/MTEP spent more time in the center of the zone compared to the zymosan group (Figure 2C). Yet no difference was noted in the total distance moved between the groups (t_(14)_ = 0.84, *p =* 0.41, Figure 2D). Taken together, the current results suggest that chronic vulvar pain may trigger anxiety and mood disturbances, potentially exacerbating pain symptoms. Conversely, regulating vulvar pain through the mGluR5 antagonist could modulate the development of anxiety-like behavior.

### 2.3. The Role of the Glutamate-mGluR5 Pathway in Vulvar Hypersensitivity Development

**Figure 3:**
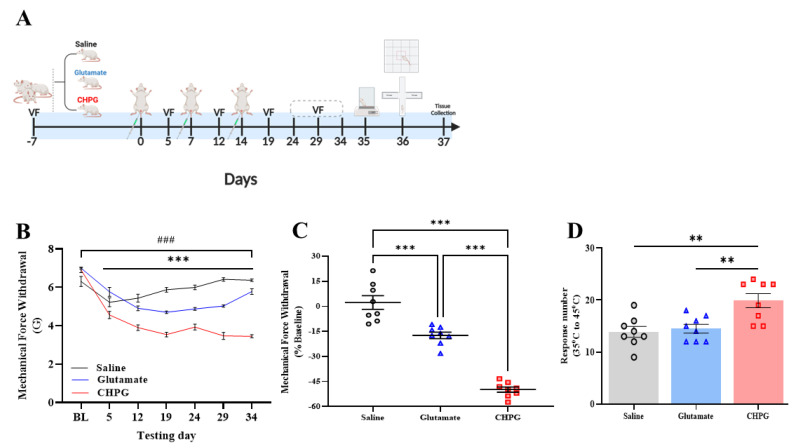
Vulvar hypersensitivity induced by a local injection of glutamate or mGluR5 agonist. (**A**) The experimental timeline and experimental procedures across the three challenges of glutamate or mGluR5-agonist CHPG. Vulvar MST was measured by Von Frey (VF). Hot plate tests, EPM tests, and open field tests were performed on day 35 and 36. (**B**) Vulvar mechanical force withdrawal (G) of the glutamate, CHPG, and saline groups. (**C**) Vulvar mechanical force withdrawal test on day 34, normalized to the baseline. (**D**) Sum of nociceptive responses in the hot plate test. One-way ANOVA and mixed model ANOVA tests were conducted, followed by Tukey’s tests. Mean ± SEM. ### *p <* 0.001 compared to baseline; ** *p <* 0.005, *** *p <* 0.001. Difference between groups.

The increase in glutamate-mGluR5 pathways after repeated vulvar inflammation challenges suggests that this pathway is involved in the development of vulvar pain. We, thus, investigated the effect of exogenous glutamate challenges, or that of the mGluR5 agonist CHPG, on vulvar mechanical and thermal hypersensitivity. We injected saline, glutamate (300 µL of 50 mM), or CHPG (300 µL of 20 mM) into the vulva. Overall, three injections were administered, with one week between each injection (Figure 3A). There was no significant long-term effect of the saline injection in the vulva on MST. Thus, the saline group displayed a significant decrease in MST only during the vulvar challenges (Figure 3B). However, in the glutamate group and the CHPG group, there was a significant decrease in MST 5 days after each challenge. Following the 2nd and the 3rd vulvar challenge, there was a significant reduction in the vulvar mechanical threshold, with no evidence of healing back to the non-allodynic levels in the CHPG group. In the glutamate group, there was a recovery to the non-allodynic levels on day 34 (i.e., 20 days after the 3rd vulvar challenge Figure 3C).

Additionally, there was a significant difference in the thermal sensitivity test among the saline, glutamate, and CHPG group [F_(2,21)_ = 8.97, *p* = 0.002, Figure 3D]. Thus, the CHPG group showed a higher thermal sensitivity response compared to the saline and glutamate groups (Figure 3D), with no significant difference between the glutamate and the saline groups (Figure 3D). The current results indicate that enhancing mGluR5 activity in the vulva can lead to mechanical and thermal vulvar hypersensitivity.

### 2.4. Vulvar Hypersensitivity Induces Anxiety Like-Behavior

**Figure 4:**
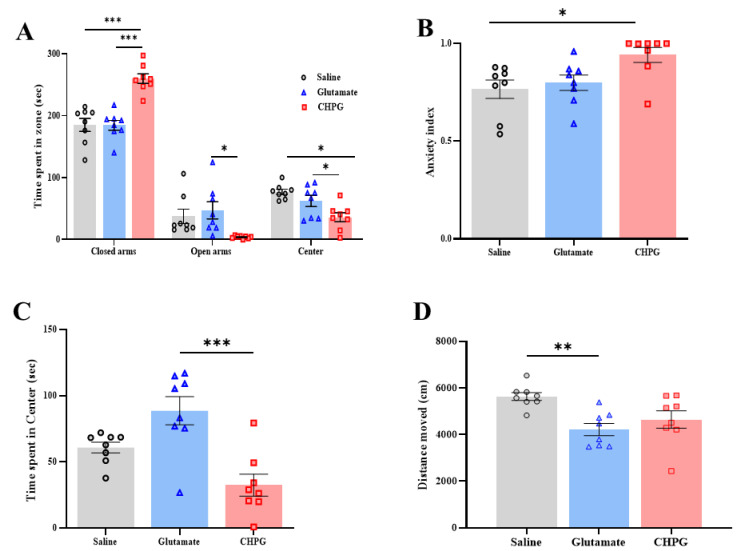
Anxiety-like behavior induced by vulvar hypersensitivity. (**A**) Time spent in the open arms, closed arms, and the center of the EPM. (**B**) Anxiety index score of the glutamate, CHPG, and saline groups (0 = low anxiety score; 1 = high anxiety score). (**C**) Time spent in the center of the open field (second). (**D**) Distance moved in the open field (cm). Behavioral assessment (*n* = 8 per group). One-way ANOVA tests were conducted, followed by Tukey’s tests. Mean ± SEM. * *p <* 0.05, ** *p <* 0.005, *** *p <* 0.001. Difference between groups.

Unsurprisingly, we observed an increased anxiety-like behavior in the CHPG group compared to the glutamate and saline groups. Accordingly, in the EPM test, the CHPG group spent more time in the closed arms compared to the saline and glutamate groups [F_(2,21)_ = 24.30, *p* < 0.001, Figure 4A]. Notably, the glutamate group spent more time in the open arms compared to the CHPG group [F_(2,21)_ = 4.730, *p* = 0.025, Figure 4A], with no significant difference between the saline and the other groups (Figure 4A). The analysis of the anxiety index revealed a significant difference among the three groups [F_(2,21)_ = 4.91, *p =* 0.018, Figure 4B]. Thus, the CHPG group showed a higher anxiety index score compared to the saline group (*p* < 0.05, Figure 4B).

In the open field test, we observed a significant difference in the time spent in the center zone of the arena among the three groups [F_(2,21)_ = 11.89, *p* < 0.001, Figure 4C]. Post-hoc analyses showed a reduction in the time spent in the center of the arena in the CHPG group compared to the glutamate group (Figure 4C), with no significant difference between the saline and the other groups. Regarding the total distance moved, there was a significant difference in the total distance moved in the arena among the three groups [F_(2,21)_ = 6.67, *p* = 0.006, Figure 4D]. Thus, the CHPG and the glutamate group tended to show, on average, a reduction in the distance moved. Yet post-hoc analyses showed a reduction in the distance moved in the glutamate group compared to the saline group (Figure 4C), with no significant difference between the saline and the CHPG groups. Unsurprisingly, the current results further support the association between vulvar pain and anxiety-like behavior in animals with chronic vulvar pain.

### 2.5. The Involvement of the Glutamate Pathway in the Neuromodulation of the Vulvar–Spinal-Cord Axis

**Figure 5:**
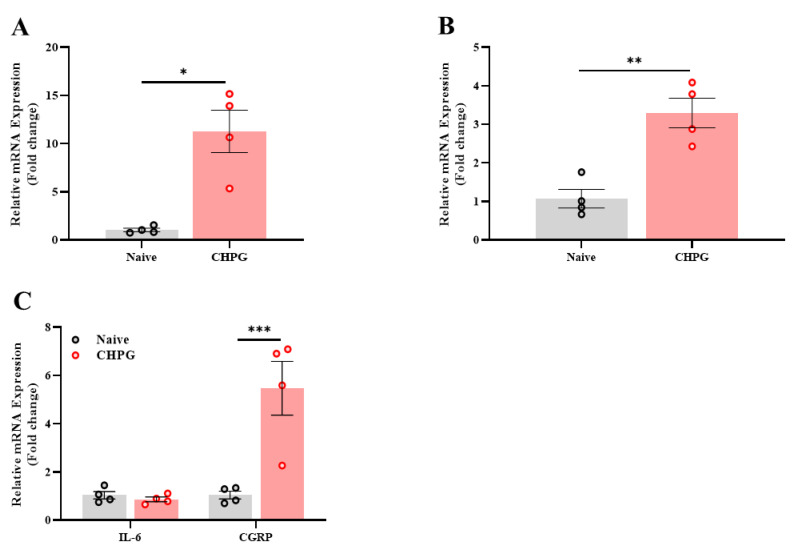
CHPG injection in the vulva increases the expression of genes related to pain channels, neuropeptides, and glutamate receptors in the sacral spine region (L6-S3). (**A**) The expression level of the pain channels TRPA1. (**B**) The expression of the glutamate receptor mGluR5. (**C**) The expression of genes related to inflammation and neuropeptides in the sacral spine region (L6-S3) after 7 days from the 3rd CHPG challenge (*n* = 4, per group). Student’s *t*-tests were performed. Mean ± SEM. * *p <* 0.05, ** *p <* 0.005, *** *p <* 0.001.

In order to further pursue the exploration of mGluR5 overactivation in the vulva, we assessed the gene expression level in the sacral nerves and spinal cord (L6-S3) of the allodynic rats in the CHPG group at the end of the experiment. We found that repeated activation of the mGluR5 in the vulva using the CHPG agonist led to a significant increase in the expression level of the TRPA channel (Figure 5A). In addition, a significant increase in the expression of the mGluR5 receptor (Figure 5B) and neuropeptide (CGRP, Figure 5C) were observed in the CHPG group compared to the naïve group.

**Figure 6:**
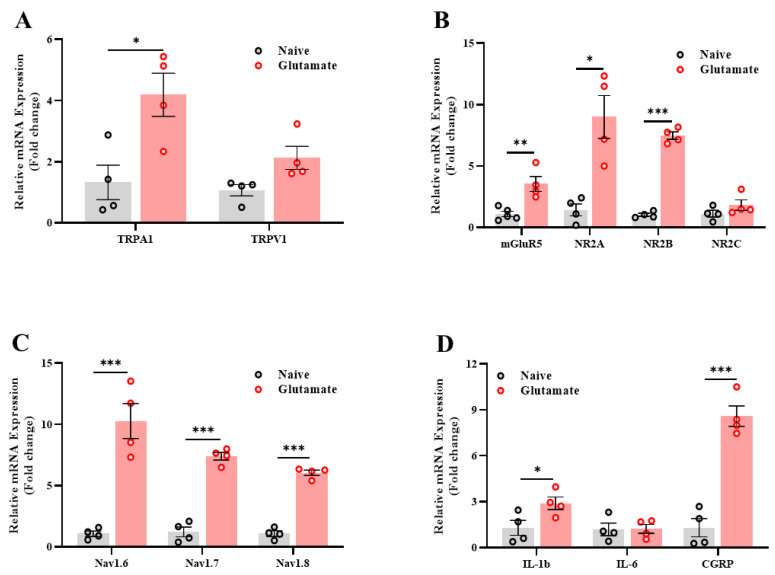
Glutamate injection in the vulva increases the expression of genes related to inflammation and neuroplasticity in the sacral spine region (L6-S3). (**A**) The expression level of the pain channels TRPA1 and TRPV1. (**B**) The expression level of the glutamate receptors mGluR5 and NMDA. (**C**) The expression of the sodium–ion channels. (**D**) The expression level of genes related to neuroinflammation and neuropeptides in the sacral spine region (L6-S3) after 7 days from the 3rd glutamate challenge (*n* = 4–5, per group). Student’s *t*-tests were conducted. Mean ± SEM. * *p <* 0.05, ** *p <* 0.005, *** *p <* 0.001.

Through further analysis, we examined the early effects of the glutamate injection in the vulva; thus, we assessed the gene expression level in the sacral nerves and spinal cord (L6-S3) 7 days after the 3rd glutamate injection in the vulva. We found that repeated glutamate injections enhanced the mRNA expression of the TRPA channel, but not that of the TRPV1 (Figure 6A). In addition, a significant increase in the expression of the mGluR5 receptor, N-methyl-D-aspartate receptor (NMDA) subunits (NR2A, NR2B, Figure 6B), sodium–ion channels (Na_V_1.6, Na_V_1.7, Na_V_1.8, Figure 6C), and pro-inflammatory cytokines and neuropeptides (IL-1β, CGRP, Figure 6D) was observed in the glutamate group compared to the naïve group.

## 3. Discussion

The current study provides evidence about the molecular and cellular mechanisms underlying the involvement of the glutamate pathway in the development of chronic vulvar pain. Previous studies suggest that inflammation-induced mGluR5 activation may play a role in the development of pain [25,33,37]. The current results support this notion. Indeed, we found that activation of the glutamate pathway by injection of exogenous glutamate, or mGluR5 agonist, in the vulva led to long-lasting vulvar mechanical and thermal hypersensitivity. Conversely, blocking mGluR5 with MTEP during the early phase of the vulvar inflammation modulated the development of vulvar mechanical and thermal hypersensitivity.

Tissue inflammation induces glutamate release from the area of the inflammation [38], resulting in pain receptor sensitization through mGluR5, which is, eventually, reflected in a decrease in the pain threshold [26,39]. Here, we examined the role of the glutamte-mGluR5 pathway in vulvar hypersensitivity development; exogenous glutamate was injected in the vulva. The current results show that repeated glutamate administration in the vulva led to mechanical allodynia that was maintained for three weeks after the 3rd glutamate challenge. However, after three weeks, the mechanical threshold of the challenged glutamate group recovered to the non-allodynic levels, but not to the baseline. Thus, a slight vulvar mechanical hypersensitivity was still observed in the glutamate group compared to the saline group.

The early effects of glutamate administration to the vulva manifested as the elevated transcription of the TRP channels (TRPV1 and TRPA1), neuropeptide and pro-inflammatory cytokines (CGRP, IL-1b), glutamate receptors (NMDA subunits: NR2A/NR2B and mGluR5), and sodium–ion channels (Na_v_1.6–1.8) in the spinal cord 7 days after the 3rd glutamate administration to the vulva, suggesting that the overactivation of the glutamate pathway in the peripheral neurons promotes neuronal changes at the transcription level, resulting in a neuronal modification that mediates peripheral hypersensitivity [40,41]. The gene expression changes were probably a result of intercellular signaling following the activation of mGluR5 in the peripheral neurons. The observed alterations in these specific genes likely represent just one aspect of the neuronal changes contributing to the development of chronic vulvar pain. Other targets, such as TRPV4 [42] and PIEZO1/2, may play an even more pivotal role in this process.

To further examine the role of the glutamte-mGluR5 pathway in vulvar hypersensitivity development, the mGluR5 agonist (CHPG) was injected in the vulva three times, with a one-week break between each injection. The current results show that specific activation of the peripheral mGluR5 led to chronic vulvar pain development. Thus, we observed a robust reduction in the vulvar mechanical threshold and an increase in thermal sensitivity that were maintained for three weeks with no evidence of recovery. Our results align with previous studies that have shown that injection of glutamate or activation mGluR5 induce hind paw and facial mechanical and thermal sensitivity [33,40]. We speculate that repeated activation of the mGluR5 in the vulva promotes gene expression adaptations in the sacral spine region, a phenomenon which possibly contributes to pain maintenance and central sensitization among the vulvar allodynic rats [43,44]. Conceivably, a single vulvar inflammation or the activation of mGluR5 increase the nociceptive signal sent from the vulva to the spinal cord [45]. Subsequently, repeated inflammation or mGluR5 activation cause a robust nociceptive signal that leads to a long-lasting increase in synaptic efficacy, for instance long-term potentiation (LTP) via the NMDA receptor, resulting in a state of reduced pain threshold [46,47]. This might explain the robust increase in NMDA receptor subunit transcription in the spinal cord among the vulvar allodynic rats that were injected with glutamate.

Another possible explanation for central sensitization may be related to pain-inhibitory neuronal loss in the spinal cord [48]. Thus, the robust increase in the Ca^2+^ influx as a result of NMDA receptor overexpression in the case of repeated inflammation challenges might trigger a mitochondria-dependent cell death pathway in GABAergic interneurons, due to the increase in mitochondrial membrane permeability [48,49]. Moreover, central sensitization and nerve hyperexcitability in the spinal cord, under inflammatory and chronic pain conditions, are strongly associated with neuroinflammation [50]. The current results align with these reports, as we found elevated transcription levels of pro-inflammatory cytokines (IL-1β, and CGRP) in the spinal cord following the glutamate injection in the vulva. This result raises the possibility that neuroinflammation may play a role in the development and maintenance of vulvar pain [51]. The putative explanation for chronic pain-induced neuroinflammation may be related to the increase in the release of glutamate and CGRP in spinal neurons as a result of Ca^2+^ and Na^+^ influx via TRP channels and NMDA receptors under chronic pain conditions, a phenomenon which can stimulate the release and production of pro-inflammatory cytokines from glial cells, resulting in long-lasting neuroinflammation [52,53].

Previous evidence suggests that blocking the mGluR5 signaling pathway during inflammation can produce anti-nociceptive effects [33,54]. Our results corroborate this evidence, as we found that blocking mGluR5 signaling in the vulva with local MTEP-mGluR5 antagonist treatment during the initial stages of inflammation partially diminished zymosan-evoked behavioral hypersensitivity, as well as vulvar allodynia development. Specifically, the vulvar mechanical threshold in 62% of the rats in the zymosan/MTEP group recovered to non-allodynic levels, with mild mechanical and thermal vulvar hypersensitivity remaining. The current results are in line with previous findings that have shown that blocking mGluR5 with the specific antagonist MTEP can modulate the development of inflammatory pain and reverse the hyperalgesia in an animal model of pain priming [36]. Regulating the activity of mGluR5 induces antinociceptive effects by modulating the intracellular signaling that contributes to pain channel sensitization in the sensory nerve terminals [33,39,55,56].

It should be noted that, in the current study, the mGluR5 antagonist treatment during inflammation did not fully regulate the development of vulvar pain. Thus, in the zymosan/MTEP group, 38% of the rats developed vulvar mechanical allodynia compared to the zymosan group. It is possible that a more effective intervention for inhibiting the mGluR5 pathway in the case of repeated inflammation requires a different concentration or treatment duration. Therefore, further research is needed to fully understand the role of the glutamate-mGluR5 signaling pathway in the development of chronic vulvar pain. In addition, combining mGluR5 antagonists with other anti-inflammatory treatments, such as mast cell inhibitors or NGF blockers, which have been implicated in the development of provoked vulvodynia (PV), may be more effective at modulating vulvar pain.

The comorbidity of chronic pain and mood disorders is quite common [57,58]. This may be associated with the fact that the underlying mechanisms of chronic vulvar pain maintenance involve central neuronal adaptations in brain networks that are responsible for pain and mood modulation [59,60,61]. Our results provide further evidence of the complex interaction between chronic vulvar pain and mental health, as we found that vulvar pain in rats causes anxiety-like behavior. This result is in line with previous evidence that has shown that women or animals with PV are more likely to experience anxiety, psychological distress, and depression [4,8,62]. This suggests that vulvar pain can trigger mood disruption and anxiety that might contribute to exacerbating the pain symptoms [63].

The current study has several limitations. The size of each group was modest, a fact which may influence the interpretation of the findings. Therefore, replications among larger samples are critical to establish the validity of the current findings. Here, we focused on investigating the role of glutamate signaling during vulvar inflammation in the development of PV. Future studies could assess whether and how enhancing the glutamate pathway during inflammation affects the development of PV.

Despite these limitations, the current findings suggest that vulvar allodynia and hypersensitivity induced by inflammation are mediated by neuroinflammation in the sacral spine region and could play a role in central sensitization and pain maintenance. Interestingly, the underlying mechanisms of PV may also involve psychological distress that contributes to pain maintenance. Critically, a more thorough examination of a specific inflammatory pathway that considers the early inflammatory events that include the upregulation of the glutamate concentration revealed that the glutamate-mGluR5 pathway is involved in the neuromodulation of vulvar neurons. Notably, reducing glutamate mGluR5 activity with the MTEP antagonist partially modulates the development of chronic vulvar pain. Thus, the current findings encourage future preclinical and clinical studies aimed at testing mGluR5 blockers’ efficacy at reducing and preventing chronic vulvar pain development.

## 4. Materials and Methods

### 4.1. Animals

Female Sprague–Dawley rats (250–300 g; 10 weeks age) were used in the present study. All animal procedures were approved by the animal care committee of Bar-Ilan University (code# BIU-MD-IL-2205-140-4, code# 84-11-2019). During the study, animals were housed in groups of three rats in a sterilized solid bottom cage with contact bedding under a controlled temperature and a 12:12 h light/dark cycle.

### 4.2. Drug Preparation

Glutamate (300 µL of 50mM SC in the vulva, Sigma-Aldrich, St. Louis, MO, USA), MTEP hydrochloride (100 µL of 10 mM SC in the vulva; cat. No. 2921, Bio-techne, Minneapolis, MN, USA), CHPG (100 µL of 10 mM SC in the vulva; cat. No. 2921, Bio-techne) [36,39], and zymosan (10 mg/mL in 300 µL SC in the vulva; cat. No. Z4250 Sigma-Aldrich, St. Louis, MO, USA) [26] were dissolved with physiological saline.

### 4.3. Vulvar Pain Model

A rat model of vulvodynia was produced using repeated zymosan-induced inflammation [26,28]. Each administered dose (10 mg/mL) contained 300 µL (1 mL syringe with a 27Gxon needle). Each rat was given three zymosan (or saline as control) injections in total, with a one-week period between each injection. The injection was carried out under 3% isoflurane anesthesia.

### 4.4. Behavioral Tests and Pain Assessment

Behavioral tests were conducted under red light illumination, following one hour of habituation in the measurement room. The EPM and OF tests were recorded and analyzed using the EthoVision XT V.15 tracking system.

#### 4.4.1. Mechanical Sensitivity

An electronic Von Frey (VF) device (cat. No. 38450 Ugo Basile) was used to assess vulvar mechanical hypersensitivity. The test was performed after thirty minutes of acclimatization in the testing chambers. A punctate stimulation was performed on the rat vulva. Five values were collected for each rat.

#### 4.4.2. Thermal Sensitivity

A hot plate analgesia meter (cat. No.3515-022 Ugo Basile) was used to assess thermal hypersensitivity. Following twenty minutes of acclimatization at a temperature of 35 °C, the plate temperature increased to 45 °C (1 °C/min). Hind paw and vulvar lickings, rearing, and jumping were documented during the experiment.

#### 4.4.3. Open Field

The animals were placed in one corner of the open field arena (L 70 × W 70 × H 50 cm). The animal activity was recorded for 10 min. Measurements of locomotor activity and time spent in the center of the arena were collected. The open field was wiped with 30% alcohol between trails.

#### 4.4.4. Elevated Plus Maze

The animals were placed in the center of the elevated plus maze. The EPM consisted of two enclosed arms (L 50 × W 10 × H 30 cm) and two open arms (L 50 × W 10 cm), and both apparatuses were elevated 40 cm above the floor. The session lasted for 5 min. The maze was wiped with 30% alcohol between trails.

### 4.5. Anxiety Index

In order to obtain comprehensive and integrated behavioral measures, the anxiety index was calculated using parameters from the elevated plus maze (EPM), including time spent and entries into the open arms, into one unified ratio with values ranging from 0 to 1, with a higher score indicating an increased anxiety level [64,65]. The following equation was used for the calculation of the anxiety index score:

(1) $$Anxiety\;Index=1-\frac{\left(\frac{Open\;arms\;time}{300sec}\right)+\left(\frac{Open\;arms\;entry}{Total\;Entries}\right)}{2}$$

### 4.6. Gene Expression Analysis

Rats were sacrificed, and the sacral nerves and the spinal cord (L6-S3) were isolated and kept at −80 °C until used. The Hybrid-R TM kit (cat. No.305-101, TAMAR, Ltd., Jerusalem, Israel) was used to extract RNA from the samples. The High-Capacity cDNA Reverse Transcription Kit (cat. No. 4368814, Thermo Fisher, Massachusetts, USA) was used to prepare the cDNA. The real-time PCR was conducted as described previously [65,66]. The relative expression of the genes was normalized to the housekeeping gene (β-actin) and calculated using the ΔΔCt method. β-actin: F- GACGTTGACATCCGTAAAGACC, R- CTAGGAGCCAGGGCAGTAATCT. CGRP: F- GTTCTCCCCTTTCCTGGTTG, R- GCTCCCTGACTTTCATCTGC. TRPA1: F- GCAGCATTTTCAGGTGCCAA, R- CGCTGTCCAGGCACATCTTA. TRPV1: F- AAGGATGGAACAACGGGCTAG, R- TCCTGGTAGTGAAGATGTGGG. IL-6: F- AAGAGACTTCCAGCCAGTTGCC, R- ACTGGTCTGTTGTGGGTGGTATC. NR2A: F- TCCATTCTTCTGTCATCCTGC, R- AAGACCGTCTCTCACTCTTGC. NR2B: F- TGCACAATTACTCCTCGACG, R- TCCGATTCTTCTTCTGAGCC. NR2C: F-GAACGGCATGATTGGGGAGGTGTA, R- CGTGTAGCTGGCGAGGAAGATGAC. mGlguR5: F- GAAAGGCCAAATAAAGGTGATCCG, R- GCGAAGATACTGGACTGGGA. IL-1b: F- AATGGACAGAACATAAGCCAACA, R- CTTCTTCTTTGGGTATTGTTTGG. Nav1.6: F- AGGATGTTAGCAGCGAATCAGACC, R- GGAGCTGGTATCGTCCAGTTTATC. Nav1.7: F- CAGCCGCAGATAGCCGTCGT, R- GGGCGTCCGCAAAGTCAGAG. Nav1.8: F- TCCTCTCACTGTTCCGCCTCAT, R- TTGCCTGGCTCTGCTCTTCATAC.

### 4.7. Data Analysis

The sample size was chosen based on our prior studies. No randomization method was used to assign animals to different experimental conditions. Statistical analyses were performed using SPSS. Student’s *t*-tests and one-way ANOVA tests were used to assess differences between groups. Mixed-model repeated-measures analysis of variance (GLM) was used to assess the changes in the mechanical threshold. Significant main effects and interactions in ANOVA and GLM tests were further pursued using the post-hoc Tukey’s test and student’s *t*-tests. The accepted significance value was set to *p* < 0.05. All data were expressed as means ± SEM. 

## Data Availability

The datasets are available from the corresponding author upon reasonable request.

## Abbreviations

TRPA1 = transient receptor potential ankyrin-1; TRPV1 = transient receptor potential vanilloid-1; GGRP = calcitonin gene-related peptide; NMDA = N-methyl-D-aspartate; PV = provoked vulvodynia; mGluR5 = metabotropic glutamate receptor 5; MTEP = 3-((2-methyl-4-thiazolyl)ethynyl)pyridine; CHPG = 2-chloro-5-hydroxyphenylglycine.
